# Macroalgae Inhibits Larval Settlement and Increases Recruit Mortality at Ningaloo Reef, Western Australia

**DOI:** 10.1371/journal.pone.0124162

**Published:** 2015-04-21

**Authors:** Fiona J. Webster, Russell C. Babcock, Mike Van Keulen, Neil R. Loneragan

**Affiliations:** 1 School of Biological Sciences and Biotechnology, Murdoch University, GPO Box S1400, Perth, W.A., 6849, Australia; 2 CSIRO Marine and Atmospheric Research, Cleveland Marine Laboratories, P.O Box 120, Cleveland, Queensland, 4163, Australia; 3 Centre for Fish, Fisheries and Aquatic Ecosystem Research, Murdoch University, GPO Box S1400, Perth, W.A., 6849, Australia; University of New South Wales, AUSTRALIA

## Abstract

Globally, many coral reefs are degraded and demonstrate reduced coral cover and increased macroalgal abundance. While negative correlations between macroalgae and coral recruitment have commonly been documented, the mechanisms by which macroalgae affects recruitment have received little attention. Here we examined the effect of macroalgae on larval settlement and the growth and survival of coral recruits, in a field experiment over nine months. Exclusion treatments were used to manipulate herbivory and macroalgal biomass, while settlement tiles measured coral settlement and survival. After nine months the volume of macroalgae was up to 40 times greater in the caged treatments than in controls and the settlement of coral larvae on the undersides of tiles within caged plots was 93% lower than in the uncaged treatments. The growth and survival of coral recruits was also severely reduced in the presence of macroalgae: survival was 79% lower in caged treatments and corals were up to 58% smaller with 75% fewer polyps. These data indicate that macroalgae has an additive effect on coral recruitment by reducing larval settlement and increasing recruit mortality. This research demonstrates that macroalgae can not only inhibit coral recruitment, but also potentially maintain dominance through a positive feedback system.

## Introduction

Coral reefs throughout the world are suffering from increased exposure to natural and anthropogenic disturbances, especially those associated with climate change [1,2]. Reefs which have suffered disturbance and coral mortality often demonstrate increased macroalgal abundance due to the ability of algae to dominant newly available space through rapid recruitment and growth [3,4]. Long term studies have shown that in healthy resilient systems corals can recover again following a decline and become dominant [5,6], however, some reefs in the world demonstrate reduced resilience and remain in a degraded state with macroalgae continuing to dominate the benthic substratum [7–11].

Coral recruitment, the product of the settlement of coral larvae and the subsequent survival of recruits to visible size, is recognized as a process critical to the maintenance and functioning of coral populations and reef ecosystems [12]. There have been a number of studies which have found a negative correlation between the macroalgal abundance and coral recruits [5,7,13–20], however the mechanisms driving these patterns and research on coral-algal interactions during the early coral life history phase has not received a lot of attention [17,21–24]. One of the reasons coral recruitment and post settlement survivorship remains little addressed is because of the challenges associated with studying coral recruits due to their small in size and tendency to settle in cryptic habitats [25].

The few studies which have focused on the effects of macroalgae on coral recruitment have found that macroalgae can affect larval behaviour, settlement and survival of recruits, however, almost all of this this research have used aquaria or in chambers, using pieces of macroalgae, typically over very short time periods i.e. < 1 week [22,26–29]. Whilst these studies provide important information about the effect of macroalgae on coral settlement and survival, the degree to which these experiments are representative of recruitment processes in a natural reef environment is unknown. Climate change projections indicate reef degradation will increase in the future [2], and given that many degraded reefs demonstrate high macroalgal cover a field based experiment examining how macroalgae affects coral recruitment is warranted. Here we investigate the role of macroalgae on coral recruitment in a nine month field experiment on the Ningaloo Reef. In particular we seek to understand the relative effects of macroalgae on coral larval settlement and post settlement survival.

The Ningaloo Reef is a fringing reef approximately 290 km in length along the central Western Australian Coast between latitudes 21° 47’ and 24° 00’ S. Coastal development is extremely low and the Ningaloo reef receives few direct human impacts compared to many other reefs, therefore, the system is considered relatively intact. The reef has been declared a marine park and has been subject to various levels of protection since the 1980’s it has also recently been listed by the IUCN as a World Heritage area. The reef has experienced limited commercial fishing activity and the main fishing pressures on the reefs come from recreational fishing [30,31]. Herbivorous species are rarely targeted [32] and therefore populations of grazing fish are mostly intact.

## Methods

### Ethics statement

This research was done in Western Australian State Waters, in accordance with permits issued by the Department of Conservation and Land Management (CE002775) and the Department of Fisheries (2007–32). All work in accordance with the code of conduct for animal ethics and approved by the animal ethics committee at Murdoch University. No protected species were sampled.

### Location and experimental treatments

All of this study took place in in a large no take zone in Coral Bay on the Ningaloo Reef, (23°07.388’S, 113°45.436’N) which has been protected since 1984. The study site was situated on the reef flat in a shallow lagoon in approximately 4m of water. The substratum was composed of limestone with low to moderate rugosity and approximately 20% cover of live coral and 40% cover of macroalgae. The urchin *Echinometra mathaei* was relatively rare at the study site (i.e. < 0.25 m^-2^).

### Effects of macroalgae on coral larval settlement

#### Manipulating macroalgal biomass

Macroalgae were manipulated using cages to exclude grazing by herbivores. Cages were constructed of steel bar and 2.5 cm plastic-coated wire mesh, with the dimensions of 0.5 x 0.5 x 0.5m and a volume of 0.125 m^3^. Cage controls consisted of two sides and a top and were oriented so cage sides faced into the current (note cage controls were not used in the larval experiment due to a lack of caging effects detected in the post-settlement experiment [33]). Uncaged plots had four steel bars in each corner only. The experimental plots were placed in areas where the substratum was characterised by limestone reef and sand, with a moderate cover of macroalgae. There were five replicates per treatment and the walls of the cages were cleaned of fouling algae on a monthly basis. The treatments for the larval settlement experiment were established in August 2006.

Macroalgal cover within the experimental treatments was measured using the point-intercept method, with a 0.5 x 0.5 m quadrat, divided into 25 equal-sized sectors with monofilament line. At the intercept of each line the benthic cover was recorded and any macroalgae identified to species level. The percentage cover of macroalgae was calculated from the number of intercepts with macroalgae divided by the total number of intercepts. The volume of macroalgae in each cage was calculated by averaging the algal height at five locations within the cage and multiplying this average height by the percent cover. For the larval settlement experiment macroalgal volume was assessed once, after eight months of caging immediately prior to the introduction of the coral larvae.

Light was measured as photosynthetically active radiation (PAR) with the sensor on the Walz Diving-Pulse Amplitude fluorometer (Walz GmbH, Effeltrich, Germany). Light was measured at the substratum level, in the caged, uncaged and partially caged plots. The measurements were taken at around 11am, and three measurements were recorded in each treatment over several minutes on a cloudless day.

#### Coral recruitment and benthic community assessment

Coral settlement was examined on limestone tiles which were 8 cm x 8 cm in dimension and 2 cm thick so the surface area on the top, bottom and vertical sides (64 cm^2^) was equal. They were drilled through the centre and attached to the substratum with a steel rod and raised from the substratum by a 2cm piece of PVC piping. Limestone tiles were conditioned in the natural reef environment for 12 months after which they were placed in caging treatments. Two limestone settlement tiles were placed in each of the treatments at the time that the cages were constructed in August 2006.

After eight months (in April 2007), when the biomass of macroalgae had increased significantly in the caged treatments, larvae of the coral *Acropora millepora* introduced to each of the treatments. The method used to collect and rear the coral larvae was similar to Babcock and Heyward [34]. On the evening of the spawning eight colonies of *Acropora millepora* were collected from the reef and placed in large plastic tubs filled with sea water, spawning occurred at around 10pm. Six of the colonies spawned in the buckets and eggs and sperm were collected from each and mixed to ensure cross fertilization. Excess sperm were removed to prevent polyspermy. Larvae were then transferred to and reared in plastic pots with mesh lids in the ocean for five days. After this time larvae were competent settle and were introduced to the treatments with a reciprocal action manual pump. To contain the larvae in the experimental treatments a mesh tent (mesh size 400 μm) was placed over each of the treatments and about 10,000 *Acropora millepora* larvae were pumped into each tent. After two days the mesh tents were removed from the experimental units, allowing sufficient time for the larvae to metamorphose and begin skeletogensis. After ten days, the tiles were collected, frozen and later examined in the laboratory under a microscope.

Coral recruitment was scored by counting the number of recruits on each of the tile surfaces. The cover of benthic organisms on each of the settlement tiles was recorded under the following broad categories: turf algae, macroalgae, CCA, and other. Turf algae, defined as <1 cm in height, included filamentous species such as *Sphacelaria*. Macroalgae defined as >1 cm in height, included larger fleshy algae such as *Lobophora variegata* and *Dictyota friabilis*. The category “other”—included invertebrates such ascidians, bryozoans, molluscs and sponges, these organisms accounted for less than 1% on the tiles and were therefore grouped together for analyses. An 8 cm x 8 cm clear plastic sheet marked with 20 randomly located points was placed over the top and bottom surfaces of the tiles and the benthic organism beneath each point noted. For the sides of tiles a 4 x 2 cm piece of plastic with 5 random points was used. Within each replicate treatment, tiles were averaged to give a mean cover of turf algae, CCA, macroalgae per tile.

Any interactions between the benthic organisms and corals were scored following protocols used by [20] where: the coral either wins (i.e. growing over) or loses an encounter (is overgrown) with another benthic organism.

#### Statistical analysis

The data for the volume of macroalgae in the treatments was not normally distributed even after transformation and therefore the non-parametric Mann Whitney t test were used to compare macroalgae in the uncaged and caged treatments. Coral larval settlement was compared between the treatments based on the number of corals recruited to the underside of the tiles and as these data were also not normally distributed comparisons were also based on the non-parametric Mann Whitney t test. The proportion of corals settling to the different tile surfaces was compared between treatments based on a two way ANOVA. The data met all assumptions of this parametric test.

The Spearman rank coefficient was used to test for correlations between the average number of recruits per tile and the volume of macroalgae, and also the relationship between irradiance and macroalgal volume. All univariate statistical analyses were conducted using SPSS Version 12.

As the underside of the tiles was the primary location of coral settlement the benthic cover on the settlement tiles was compared for the undersurfaces only using the multivariate statistic PERMANOVA. For the PERMANOVA analyses data were fourth root transformed and comparisons were based on Bray Curtis using a fixed design. Multivariate analyses were performed using Primer-E v6 software [35] with the PERMANOVA+ add-on package [36].

### Effects of macroalgae on the growth and survival of coral recruits

#### Manipulating macroalgal biomass

For the post settlement experiment cages, cage controls and uncaged plots were used similar to described for the larval settlement experiment. There were a minimum of three replicates per treatment. The volume of macroalgae in the treatments was assessed when cages were constructed in October 2004, again five months later when the settlement tiles were introduced (March 2005) and thereafter every three months until the conclusion of the experiment in December 2005.

#### Coral recruitment and benthic community assessment

Settlement tiles were conditioned in the different caging treatments (caged, uncaged and partial cage) for five months before being seeded with *Acropora millepora* recruits. Whilst some macroalgae had developed on the settlement tiles, the majority was attached to the reef benthos within the treatments. To remove the potential effects of macroalgal canopy on coral settlement, and ensure there were adequate densities of recruits for the long term experiment, all tiles were removed from the different treatments for larval settlement and placed in benthic chambers on the ocean floor for coral seeding. The tiles were not cleaned so any benthic communities on the tiles which had developed over the five month period were undamaged. The chambers were rectangular containers (20 L volume) with large openings cut out of the sides and lid, which were sealed with 250 μm plankton mesh to permit water exchange but prevent the larvae from escaping. Five tiles were placed in each container in a horizontal position (as per previous conditioning) on a stainless steel screw, so that each tile was raised approximately 2 cm above the bottom of the container. Approximately 3,000 *Acropora millepora* larvae were injected into the containers, so approximately 600 larvae were available to settle per tile. After two days, the tiles were returned to their original field treatments of caged, uncaged and partially caged plots. Two tiles were placed in each replicate treatment.

The growth and survival of the *A*. *millepora* recruits was assessed by removing the tiles from the field and taking them to a field laboratory for examination under a microscope in a bath of seawater. The first assessment of coral recruits on the settlement tiles was ten days after the tiles were introduced to the cages. Subsequent assessments of coral recruit growth and survival were conducted every three months up until nine months when there were insufficient numbers of recruits to continue the experiment. Initially corals were counted on all surfaces, but because settlement was predominantly on the underside, only this surface was examined in subsequent assessments.

The benthic communities on all surfaces of the limestone settlement tiles were assessed under a microscope in a bath of seawater using the same technique as described in the larval settlement experiment. Benthic communities were assessed once prior seeding the tiles with coral recruits.

#### Statistical analysis

Between treatment changes in the volume of macroalgae through time was compared using Permutational Analysis of Variance (PERMANOVA). The data were not normally distributed however the PERMANOVA method (which determines statistical significance by permutation), makes no a priori assumptions about the distribution of the data. Data were fourth root transformed and comparisons of changes in the volume of macroalgae through time were based on a Euclidian distance matrix. In the PERMANOVA design, time was designated as a random effect.

Assessments of coral settlement, growth and survival were all based on corals recruited to the undersides of the tiles as this was the primary location of settlement. To test if there were any significant differences in the number of recruits initially settling on the tiles in the different treatments a one way ANOVA was performed. The proportion of corals settling to the different tile surfaces was compared between treatments based on a two way ANOVA. The survival of coral recruits through time was assessed by tracking each individual recruit and survivorship was assessed using the Kaplan Meier Survival Analysis.

Coral recruit growth was assessed by measuring the longest linear dimension and counting the number of polyps per coral. Coral growth was assessed as the average growth of each coral recruit in the treatments and Repeated Measures ANOVA ‘s were used to statistically compare growth in different treatments over the nine month period. The Spearman rank co-efficient was used to assess the relationships between macroalgal volume and coral growth and survival.

## Results

### Effect of macroalgae on coral larval settlement

#### Macroalgae in cages

**Fig 1:**
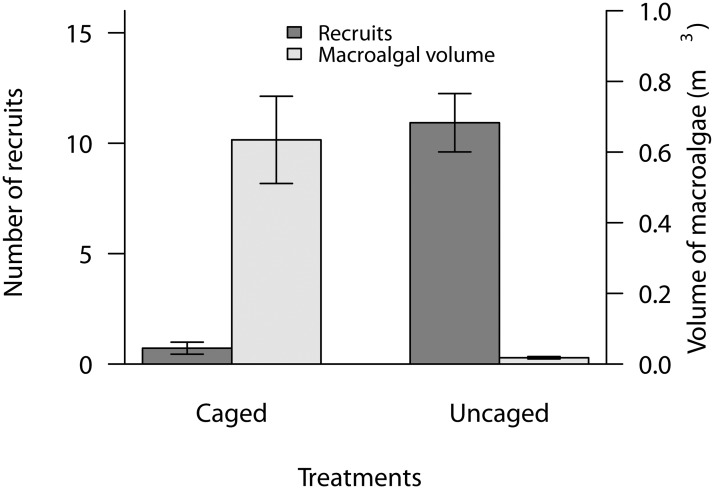
Average volume of macroalgae and number of coral recruits on settlement tiles in the caged and uncaged treatments in the larval settlement experiment.

The volume of macroalgae was almost 40 times higher in the caged treatments than the uncaged treatments (Fig 1a, t = -2.61, p < 0.01). The caged plots were dominated by bushy, upright *Sargassum* spp., which formed a thick canopy. In contrast, the low volume of macroalgae in uncaged treatments consisted of prostrate species such as *Dictyota friabilis* and *Lobophora variegata*.

#### Benthic cover on the tiles

In the larval settlement experiment the benthic cover on the underside of the settlement tiles was mostly “bare” (covered with a biofilm of diatoms) and CCA (S1 Table). There were no significant differences for the percentage cover (PERMANOVA F = 1.76, p = 0.24, S2 Table) of the different benthic assemblages on the settlement tiles between the treatments.

#### Settlement of coral larvae

Coral settlement was severely reduced beneath the macroalgal canopy (by approximately 93%) and there were significant differences in the number of recruits on the undersides of tiles in the caged versus uncaged treatments (Fig 1b, t = 2.62, p < 0.01).

There was very little recruitment to the upper tile surfaces in both treatments. In the caged treatment there was a greater tendency for corals to settle on the vertical surfaces, and less of a tendency to settle to the under surfaces. In comparison in the uncaged treatment all most all settlement was on the under surface, (S3 Table), these differences, however were not significant (F = 0.57, p = 0.56, S4 Table).

The volume of macroalgae was negatively correlated with the number of coral recruits (r = -0.80, p < 0.005) and light levels (PAR) (r = -0.87, p < 0.01). The PAR at the substratum was 90% lower in the caged than the uncaged treatments (17.0 ± 9.9 S.E and 162.5 ± 2.2 S.E μmol photon m^-2^ s^-1^ respectively).

### Effects of macroalgae on the growth and survival of coral recruits

#### Macroalgae in cages

**Fig 2:**
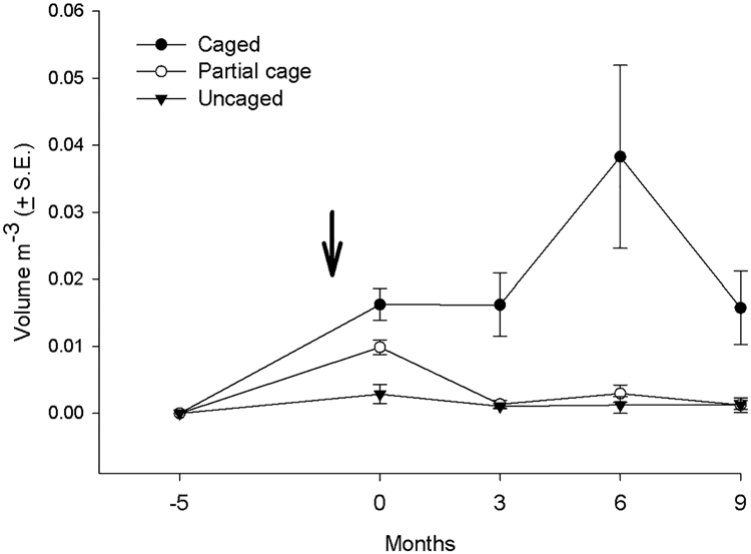
Average volume of macroalgae in the different treatments (uncaged, partially caged and caged) in the post settlement experiment over a 14 month period. Note that coral recruits were introduced to the treatments at time 0.

The mean volume of macroalgae increased significantly in the caged plots through time, but remained low in the uncaged and partially caged plots (Fig 2, Table 1 and S5 Table). The mean volume in the cages was highest after six months due to the seasonal growth of *Hydroclathrus clathratus*, which disappeared almost completely during the subsequent three months. At the conclusion of the experiment the caged treatments were dominated by the bushy upright *Sargassum* spp. and *Dictyota cervicornis*.

**Table 1:** Results of PERMANOVA and two way ANOVA’s comparing the volume of macroalgae and coral size in caged, uncaged cage controls in the post settlement experiment.

	Macroalgae		Size (length)		Size (polyps)	
	F	p	F	p	F	p
Treatment	21.81	0.002	6.63	0.02	10.88	0.005
Time	3.54	0.009	60.43	0.0001	60.78	0.0001
Treatment x Time	3.12	0.004	7.56	0.000	8.81	0.0001

#### Benthic cover on settlement tiles

In the post-settlement experiment the benthic cover on the underside of tiles was assessed at the start of the experiment, immediately prior to the tiles being seeded by coral larvae. The main benthic cover was bare “bare” (covered with a biofilm of diatoms) and turf algae (S6 Table). There were no significant differences in the benthic cover on the tiles between the caged, uncaged and partially caged treatments (PERMANOVA F = 0.96, p = 0.47, S7 Table).

#### Larval settlement

The total number of corals present on the underside of tiles at the start of the post-settlement experiment did not differ significantly among treatments (F = 1.05, p = 0.38, S8 Table). There was an average of 57.2 ± 11.3 S.E coral recruits per tile at the start of the experiment. The majority of corals settled to the undersurface of the tiles (S9 Table) and there were no significant differences in the proportion of corals settling to the different tiles surfaces between treatments (F = 0.78, p < 0.54, S10 Table)

#### Coral recruit survival

**Fig 3:**
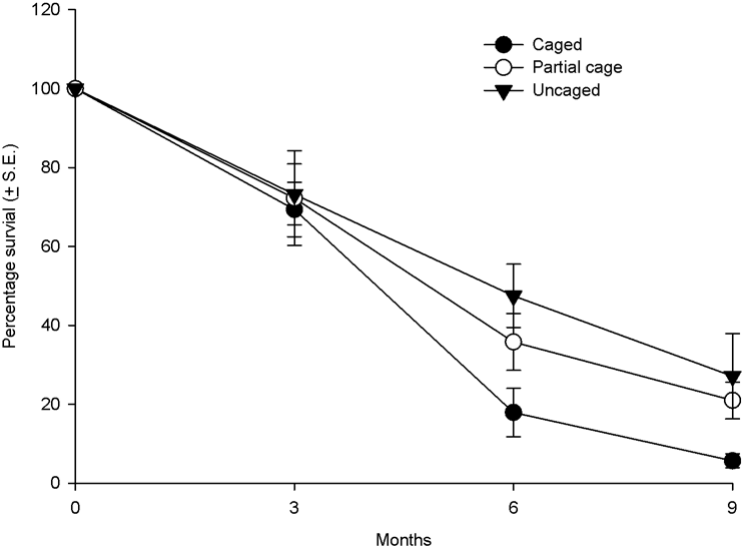
Average survival of coral recruits in the different treatments (uncaged, partially caged and caged) over a nine month period in the post settlement experiment.

Over the nine month period, the number of surviving corals decreased in all treatments, however, the survival was much lower in the caged treatments compared to the uncaged treatments and cage controls (Fig 3). By the conclusion of the experiment the percentage of surviving corals was on average, 5.7 + 1.7 S.E. % in the caged treatments compared to 27.1 + 4.6 S.E. % in the uncaged treatments. Proportionally survival was 79% lower in the caged compared to the uncaged treatments by the experiment end. Statistical tests demonstrated lower survival through time in caged treatments (*S*_*t*_ = 35.97, p < 0.001), with post hoc analyses identifying significant differences in post settlement survival among all treatments (S11 Table).

There were only 16 observations of direct overgrowth of coral recruits (about 1%) throughout the experiment, mainly by bryozoans and sponges. The survival of recruits was negatively correlated with macroalgal volume at the conclusion of the experiment (r = -0.72, p < 0.01).

#### Coral recruit growth

**Fig 4:**
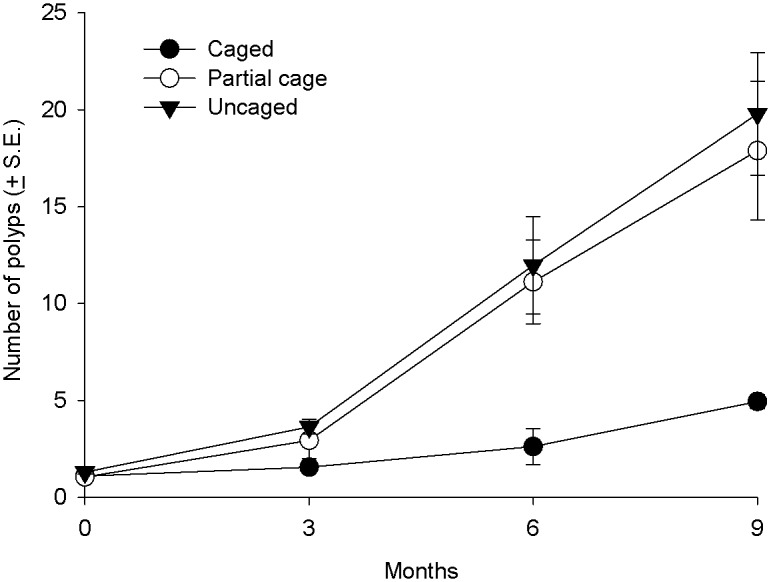
Average growth (number of polyps) of coral recruits in the different treatments (uncaged, partially caged and caged) over a nine month period in the post settlement experiment.

At the start of the experiment, all coral recruits consisted of one polyp and had a similar maximum linear dimension of around 0.7 mm. At the conclusion of the experiment corals in caged treatments were significantly smaller than those in the uncaged treatments and cage controls (p < 0.0001). Coral recruits in the caged treatments had on average 75% fewer polyps (4.9 ± 0.3 S.E) than corals in the uncaged treatments (20.0 ± 3.2 S.E) and cage controls (17.9 ± 3.57 S.E) (Fig 4, Table 1 and S5 Table). Similarly, the maximum linear diameter of corals in the caged treatments (13.6 ± 1.2 mm) was 58% less than the uncaged treatments (32.6 ± 4.6 S.E mm) and control (31.3 ± 6.8 S.E mm) (Table 1 and S5 Table).

There was a significant negative correlation between macroalgal volume and maximum linear width of corals at the conclusion of the experiment (r = -0.39, p < 0.01).

## Discussion

Larval settlement was severely reduced by in the presence of macroalgae, with 93% fewer larval recruits on the settlement tiles beneath the macroalgal canopy compared to open plots. This significant reduction in recruitment suggests that the larvae preferred to keep swimming rather than settle in habitats which are unsuitable for survival. As larvae have limited energy reserves and are poor swimmers [37] these results indicate that in areas of high macroalgal abundance most larvae will not settle but rather continue to search with further exposure to starvation, predation and environmental stress [38,39]. Larvae which did chose to settle beneath the macroalgal canopy did so at their peril.

The growth and survival of the coral recruits was severely compromised by the presence of macroalgae. After nine months, coral recruits were on average 58% smaller (max linear length) and had 75% fewer polyps than recruits which grew on settlement tiles in the open plots. Rapid growth in coral recruits is critical for competition with other sessile organisms [29] and also so that minor damage does not result in the mortality of the whole colony [40]. Size is also known to have a critical influence on survivorship of coral recruits, with the probability of survival increasing with recruit size even without the effects of competition and predation [25,41]. We found that survival of coral recruits was severely compromised beneath the macroalgal canopy, with 79% lower survival compared to uncaged treatments after a nine month period. The effect of macroalgae on coral survival became more pronounced through time, with significant differences manifesting after three months, which is when significant size differences between treatments also became apparent. When considered in combination, the effects of macroalgae on coral larval settlement and recruit mortality were devastating, with the chances of a coral successfully recruiting and surviving to the nine month stage being 98.5% lower in the presence of macroalgae.

Light was found to be 90% lower beneath the macroalgal canopy and this is likely to have played a significant role in reduced larval settlement, coral growth and survival in the caged treatments. Reduced light would have lowered photosynthesis by the zooxanthallae and the supply of essential compounds provided to the coral host for calcification, growth and repair [42,43]. Other research has found that coral settlement and survival can be strongly influenced by other benthic organisms such as turf algae (i.e. <1 cm in height) [44,45] and sponges [46]. In our experiments the development of benthic communities on the undersides of the tiles was relatively low, and was either bare (i.e. thin layer of diatoms and sparse turf algae) or CCA. Consequently space monopolisation and competition between coral recruits and other benthic organisms was minimal with <1% of coral recruits in direct contact with other organisms. Predation and incidental grazing by parrot fish can also play a role in coral recruit mortality, [47,48], however, our study found no effects as almost all corals recruited to the undersides of tiles where they were protected.

There are other several mechanisms by which macroalgae may have inhibited the settlement, growth and survival of coral recruits including: effects of microbes [24,49] and allelochemicals [22,26], reduced oxygen exchange [50], uptake of nutrients [51] and lowered encounter rates with food particles [52] and abrasion [53]. Different water chemistry in the settlement experiment where treatments were encased in a mesh tent, also may have played a role. Whilst the mesh tents allowed water exchange—it is likely that the treatments differed in terms of CO_2_, O_2_ and potentially allelopathic chemicals and bacterial communities due to the higher biomass of macroalgae in the caged treatments.

Reduced larval settlement and survival in the presence of macroalgae has also been observed in other research [22,26,27], but our results differ in the magnitude and timing of effects. These differences are likely to be attributed to the current study being a long term field based experiment whereas previous research involved aquaria or chambers, over relatively short time periods using either pieces of macroalgae or extracts. Coral larval settlement was more strongly inhibited in our studies than others, probably because our research was conducted in cages with a full canopy of macroalgae whereas other experiments which used only small macroalgal pieces [22,26,27]. In contrast, in our experiments, macroalgae had less of an effect on recruit survival in the short term. Other experiments demonstrated reduced coral survival in the presence of macroalgae within 1 week [26] whereas in our research significant effects only became apparent after six months. We are unsure why we did not see a significant reduction in coral growth and survival in the shorter term, perhaps the recruits were also affected by other external factors (e.g. temperature) which counteracted any macroalgal effects. At six months however, growth and survival decreased significantly inside the caged treatments, in concurrence with a large seasonal growth of macroalgae.

Our experiments demonstrate that by inhibiting coral recruitment, growth and survival macroalgae can create re-enforcing feedbacks, which has important consequences for reefs recovering from disturbance. Other potential mechanisms by which macroalgae could maintain dominance include coral poisoning [54], reduced coral reproduction [55,56] and further reduced grazing by herbivores [57]. All of these mechanism could create a bottle neck in coral recruitment and facilitate the continued stability and expansion of macroalgae and prevent the return of the reef to a coral dominated system [58].

Whilst it is clear that high levels of macroalgae are a characteristic of degraded reefs [59–61] the presence of macroalgae does not always signify degradation and maybe a natural characteristic of some coral reef ecosystems [62–64]. The Ningaloo Reef has a mosaic of reefs types, with some areas characterised by large areas of macroalgae and others coral dominated [65,66]. Human influences on the Ningaloo Reef are relatively low compared to compared to other reefs in the world and the areas of macroalgal dominance are thought to be related to low reef rugosity and lack of protective habitat for herbivore populations [67]. Positive feedback mechanisms are also likely to play a role in maintaining the macroalgal patch mosaic on the Ningaloo Reef.

It is predicted that coral reef disturbances from natural and anthropogenic influences will increase in the future [2]. The ability of coral communities to recover from disturbance is critical to their long-term persistence and is dependent on both the ongoing replenishment of coral populations through larval recruitment, as well as the maintenance of suitable substrates for coral settlement and growth [16]. This study has highlighted the ability of macroalgae to suppress coral recruitment thereby inhibiting recovery of coral reefs from disturbance. From a management perspective, maintaining herbivores to limit macroalgal expansion and reef overgrowth is critical for coral reef resilience.

## Supporting Information

S1 Table Benthic cover on the under surface of the settlement tiles for the coral larval settlement experiment. (DOCX)

S2 Table PERMANOVA results- comparison of benthic cover on the under surface of settlement tiles between the caged and uncaged treatments in the coral larval settlement experiment. (DOCX)

S3 Table Average percentage of corals (S.E) settling on the top, sides and bottom of the settlement tiles in the coral larval settlement experiment. (DOCX)

S4 Table Results of a two way ANOVA comparing the different proportions of corals settling on the different settlement tile surfaces (bottom, sides and top) in the coral larval settlement experiment. (DOCX)

S5 Table Results of repeated measures ANOVA for the volume of macroalgae and size of coral recruits in the post settlement experiment through time. Note that the analysis of macroalgal volume was undertaken using PERMANOVA.
(DOCX)

S6 Table Benthic cover on the under surface of the settlement tiles for the post settlement experiment. (DOCX)

S7 Table PERMANOVA results- comparison of benthic cover on settlement tiles between the caged, uncaged and partially caged in the post settlement experiment. (DOCX)

S8 Table One way ANOVA comparing the number of corals settling on the underside of tiles at the start of the post settlement experiment. (DOCX)

S9 Table Average percentage of corals (S.E) settling on the top, sides and bottom of the settlement tiles in the post settlement experiment. (DOCX)

S10 Table Results of a two way ANOVA comparing the different proportions of corals settling on the different settlement tile surfaces (top, sides and bottom) in the post settlement experiment. (DOCX)

S11 Table Pairwise multiple comparison procedures (Holm-Sidak method) for the Kaplan Meier Survival Analysis for the post settlement experiment. (DOCX)
