# Supplementary material for: Macroalgae Inhibits Larval Settlement and Increases Recruit Mortality at Ningaloo Reef, Western Australia
Source: PLoS One. 2015 Apr 21;10(4):e0124162. doi: 10.1371/journal.pone.0124162 (PMC4405272; doi:10.1371/journal.pone.0124162)
Supplement: S1 Table — (DOCX) [file pone.0124162.s001.docx]

# Supporting Information

**S1 Table. Benthic cover on the under surface of the settlement tiles for the coral larval settlement experiment**

|  | **Bare** | **CCA** | **Macroalgae** | **Turf algae** | **Other** |
| --- | --- | --- | --- | --- | --- |
| Caged | 62.7 ± 6.8 | 33.4 + 6.5 | 2.2 + 1.2 | 0.4 + 0.4 | 1.3 + 1.2 |
| Uncaged | 58.9 + 5.5 | 32.1 + 5.6 | 6.6 + 2.1 | 2.4 + 1.4 | 0 + |
