# Supplementary material for: Macroalgae Inhibits Larval Settlement and Increases Recruit Mortality at Ningaloo Reef, Western Australia
Source: PLoS One. 2015 Apr 21;10(4):e0124162. doi: 10.1371/journal.pone.0124162 (PMC4405272; doi:10.1371/journal.pone.0124162)
Supplement: S2 Table — (DOCX) [file pone.0124162.s002.docx]

# Supporting Information

**S2 Table. PERMANOVA results- comparison of benthic cover on the under surface of settlement tiles between the caged and uncaged treatments in the coral larval settlement experiment**

|  | **df** | **MS** | **Pseudo F** | **p (perm)** |
| --- | --- | --- | --- | --- |
| Treatment | 1 | 298.68 | 1.76 | 0.24 |
| Error | 9 | 170.12 |  |  |
| Total | 10 |  |  |  |
