# Supplementary material for: Macroalgae Inhibits Larval Settlement and Increases Recruit Mortality at Ningaloo Reef, Western Australia
Source: PLoS One. 2015 Apr 21;10(4):e0124162. doi: 10.1371/journal.pone.0124162 (PMC4405272; doi:10.1371/journal.pone.0124162)
Supplement: S3 Table — (DOCX) [file pone.0124162.s003.docx]

# Supporting Information

**S3 Table. Average percentage of corals (S.E) settling on the top, sides and bottom of the settlement tiles in the coral larval settlement experiment**

| **Treatment** | **Position on tile** | | |
| --- | --- | --- | --- |
|  | **Top** | **Sides** | **Bottom** |
| Caged | 0.0 ± 0.0 | 45.3 ± 19.7 | 54.7 ± 19.7 |
| Uncaged | 2.6 ± 1.3 | 26.5 ± 4.2 | 70.9 ± 5.2 |
