# Supplementary material for: Macroalgae Inhibits Larval Settlement and Increases Recruit Mortality at Ningaloo Reef, Western Australia
Source: PLoS One. 2015 Apr 21;10(4):e0124162. doi: 10.1371/journal.pone.0124162 (PMC4405272; doi:10.1371/journal.pone.0124162)
Supplement: S4 Table — (DOCX) [file pone.0124162.s004.docx]

# Supporting Information

**S4 Table. Results of a two way ANOVA comparing the different proportions of corals settling on the different settlement tile surfaces (bottom, sides and top) in the coral larval settlement experiment**

|  | **df** | **MS** | **F** | **p** |
| --- | --- | --- | --- | --- |
| Treatment | 1 | 0.06 | 0.37 | 0.55 |
| Orientation | 2 | 1.51 | 8.99 | 0.001 |
| Treatment * Orientation | 2 | 0.09 | 0.57 | 0.56 |
| Error | 24 | 0.07 |  |  |
| Total | 30 |  |  |  |
