# Supplementary material for: Macroalgae Inhibits Larval Settlement and Increases Recruit Mortality at Ningaloo Reef, Western Australia
Source: PLoS One. 2015 Apr 21;10(4):e0124162. doi: 10.1371/journal.pone.0124162 (PMC4405272; doi:10.1371/journal.pone.0124162)
Supplement: S5 Table — Note that the analysis of macroalgal volume was undertaken using PERMANOVA. (DOCX) [file pone.0124162.s005.docx]

# Supporting Information

**S5 Table. Results of repeated measures ANOVA for the volume of macroalgae and size of coral recruits in the post settlement experiment through time. Note that the analysis of macroalgal volume was undertaken using PERMANOVA**

|  | **df** | **SS** | **MS** | **F** | **p** |
| --- | --- | --- | --- | --- | --- |
| Macroalgae |  |  |  |  |  |
| Treatment | 2 | 68.20 | 34.10 | 68.12 | 0.001 |
| Time | 5 | 8.88 | 1.78 | 3.55 | 0.006 |
| Treatment x Time | 10 | 15.64 | 1.57 | 3.12 | 0.004 |
| Error | 54 | 27.03 | 0.05 |  |  |
|  |  |  |  |  |  |
| Size (# polyps) |  |  |  |  |  |
| Treatment | 2 | 320.48 | 160.24 | 10.88 | 0.005 |
| Time | 3 | 1241.82 | 413.94 | 60.78 | 0.000 |
| Treatment x Time | 6 | 360.13 | 60.02 | 8.81 | 0.000 |
| Error | 24 | 163.44 | 6.81 |  |  |
|  |  |  |  |  |  |
| Size (Length mm) |  |  |  |  |  |
| Treatment | 2 | 620.11 | 310.06 | 6.63 | 0.02 |
| Time | 3 | 2641.39 | 880.46 | 60.43 | 0.000 |
| Treatment x Time | 6 | 660.91 | 110.15 | 7.56 | 0.000 |
| Error | 24 | 349.65 | 14.57 |  |  |
