# Supplementary material for: Macroalgae Inhibits Larval Settlement and Increases Recruit Mortality at Ningaloo Reef, Western Australia
Source: PLoS One. 2015 Apr 21;10(4):e0124162. doi: 10.1371/journal.pone.0124162 (PMC4405272; doi:10.1371/journal.pone.0124162)
Supplement: S6 Table — (DOCX) [file pone.0124162.s006.docx]

# Supporting Information

**S6 Table. Benthic cover on the under surface of the settlement tiles for the post settlement experiment**

|  | **Bare** | **CCA** | **Macroalgae** | **Turf algae** | **Other** |
| --- | --- | --- | --- | --- | --- |
| Uncaged | 61.8 + 4.6 | 6.1 + 3.5 | 7.4 + 4.5 | 23.6 + 5.2 | 1.1 + 0.7 |
| Partial cage | 65.1 + 6.7 | 5.1 +3.1 | 9.4 + 3.2 | 19.9 + 3.9 | 0.5 + 0.5 |
| Caged | 63 + 7.1 | 6.1 + 4.3 | 6.4 + 3.7 | 21.4 + 4.2 | 3.1 + 1.7 |
