# Supplementary material for: Macroalgae Inhibits Larval Settlement and Increases Recruit Mortality at Ningaloo Reef, Western Australia
Source: PLoS One. 2015 Apr 21;10(4):e0124162. doi: 10.1371/journal.pone.0124162 (PMC4405272; doi:10.1371/journal.pone.0124162)
Supplement: S7 Table — (DOCX) [file pone.0124162.s007.docx]

# Supporting Information

**S7 Table. PERMANOVA results- comparison of benthic cover on settlement tiles between the caged, uncaged and partially caged in the post settlement experiment**

|  | **df** | **MS** | **Pseudo F** | **p (perm)** |
| --- | --- | --- | --- | --- |
| Treatment | 2 | 324.92 | 0.95711 | 0.47 |
| Residual | 13 | 339.48 |  |  |
| Total | 15 |  |  |  |
