# Supplementary material for: Macroalgae Inhibits Larval Settlement and Increases Recruit Mortality at Ningaloo Reef, Western Australia
Source: PLoS One. 2015 Apr 21;10(4):e0124162. doi: 10.1371/journal.pone.0124162 (PMC4405272; doi:10.1371/journal.pone.0124162)
Supplement: S8 Table — (DOCX) [file pone.0124162.s008.docx]

# Supporting Information

**S8 Table. One way ANOVA comparing the number of corals settling on the underside of tiles at the start of the post settlement experiment**

|  | **df** | **MS** | **F** | **p** |
| --- | --- | --- | --- | --- |
| Between groups | 2 | 2008.69 | 1.05 | 0.38 |
| Within groups | 13 | 1910.79 |  |  |
| Total | 15 |  |  |  |
