# Supplementary material for: Macroalgae Inhibits Larval Settlement and Increases Recruit Mortality at Ningaloo Reef, Western Australia
Source: PLoS One. 2015 Apr 21;10(4):e0124162. doi: 10.1371/journal.pone.0124162 (PMC4405272; doi:10.1371/journal.pone.0124162)
Supplement: S9 Table — (DOCX) [file pone.0124162.s009.docx]

# Supporting Information

**S9 Table. Average percentage of corals (S.E) settling on the top, sides and bottom of the settlement tiles in the post settlement experiment**

| **Treatment** | **Position on tile** | | |
| --- | --- | --- | --- |
|  | **Top** | **Side** | **Bottom** |
| Caged | 2.76 + 2.38 | 5.99 + 1.59 | 91.25 + 3.59 |
| Control | 0.22 + 0.14 | 2.70 + 1.32 | 97.09 + 1.44 |
| Uncaged | 1.91 + 1.81 | 7.84 + 7.76 | 90.23 + 6.52 |
