# Supplementary material for: Macroalgae Inhibits Larval Settlement and Increases Recruit Mortality at Ningaloo Reef, Western Australia
Source: PLoS One. 2015 Apr 21;10(4):e0124162. doi: 10.1371/journal.pone.0124162 (PMC4405272; doi:10.1371/journal.pone.0124162)
Supplement: S10 Table — (DOCX) [file pone.0124162.s010.docx]

# Supporting Information

**S10 Table. Results of a two way ANOVA comparing the different proportions of corals settling on the different settlement tile surfaces (top, sides and bottom) in the post settlement experiment**

|  | **df** | **MS** | **F** | **p** |
| --- | --- | --- | --- | --- |
| Treatment | 2 | 0.11 | 0.29 | 0.79 |
| Orientation | 2 | 16.09 | 424.26 | 0.0001 |
| Treatment * Orientation | 4 | 0.029 | 0.778 | 0.54 |
| Error | 87 | 0.39 |  |  |
| Total | 96 |  |  |  |
