# Supplementary material for: Macroalgae Inhibits Larval Settlement and Increases Recruit Mortality at Ningaloo Reef, Western Australia
Source: PLoS One. 2015 Apr 21;10(4):e0124162. doi: 10.1371/journal.pone.0124162 (PMC4405272; doi:10.1371/journal.pone.0124162)
Supplement: S11 Table — (DOCX) [file pone.0124162.s011.docx]

# Supporting Information

**S11 Table. Pairwise multiple comparison procedures (Holm-Sidak method) for the Kaplan Meier Survival Analysis for the post settlement experiment**

|  | **Statistic** | **p** |
| --- | --- | --- |
| Uncaged versus Caged | 35.97 | 0.0001 |
| Control versus Uncaged | 6.40 | 0.00229 |
| Control versus Caged | 4.62 | 0.0315 |
